# Clinical Spectrum and Dynamics of Sequelae Following Tick-Borne Encephalitis Virus Infection: A Systematic Literature Review

**DOI:** 10.1093/ofid/ofaf317

**Published:** 2025-05-29

**Authors:** Kate Halsby, Liesl Gildea, Pingping Zhang, Frederick J Angulo, Andreas Pilz, Jennifer Moisi, Ann Colosia, Johann Sellner

**Affiliations:** Global Vaccines and Anti-Infectives Medical Affairs, Pfizer, Surrey, United Kingdom; Value and Access, RTI Health Solutions, Manchester, United Kingdom; Medical Affairs Evidence Generation Statistics, Pfizer Research and Development, Collegeville, Pennsylvania, USA; Global Vaccines and Anti-Infectives Medical Affairs, Pfizer, Collegeville, Pennsylvania, USA; Global Vaccines and Anti-Infectives Medical Affairs, Pfizer, Vienna, Austria; Global Vaccines and Anti-Infectives Medical Affairs, Pfizer, Paris, France; Value and Access, RTI Health Solutions, Durham, North Carolina, USA; Department of Neurology, Landesklinikum Mistelbach-Gänserndorf, Mistelbach, Austria affiliated with Karl Landsteiner University of Health Sciences, Krems, Austria; Department of Neurology, Christian Doppler Medical Center, Paracelsus Medical University, Salzburg, Austria; Department of Neurology, Klinikum Rechts der Isar, School of Medicine, Technische Universität München, München, Germany

**Keywords:** clinical manifestations, long-term outcome, nervous system damage, sequelae, tick-borne encephalitis

## Abstract

**Background:**

Infection with the tick-borne encephalitis virus (TBEV) can affect the nervous system and lead to significant morbidity. To summarize current knowledge of long-term outcomes following TBEV infection, we systematically reviewed the prevalence of TBEV infection sequelae after hospital discharge across different age groups and follow-up time points.

**Methods:**

Studies of adults, children, and “all-age” populations with laboratory-confirmed TBEV infection were identified via electronic database searches. Study categorization was based on follow-up time after hospital discharge: ≤6, 7 to ≤12, or >12 months. Sequelae signs/symptoms were divided into 3 categories: neurological, neuropsychiatric, and other. Data were normalized using weighted means. Heterogeneity was estimated using a meta-analytic random-effects model.

**Results:**

Fifteen studies were eligible for analysis (13 included only hospitalized patients). Seventy-nine unique sequelae symptoms were identified. Adults had a higher frequency of persistent symptoms than children (20.6%–100% vs 1.7%–69%). There were high levels of data heterogeneity (*I*^2^ > 90%) among all studies. Although the proportion of patients with each sequela fluctuated across time, headache was reported by ≥20% of patients at all time points. Some sequelae also varied by age group; for example, irritability was more frequent in children, while insomnia/sleep disorders were more frequent in adults. Predominant neurological symptoms included balance disorders and headache. Predominant neuropsychiatric symptoms included concentration and memory disorders.

**Conclusions:**

Patients experience a variety of neurological, neuropsychiatric, or other sequelae symptoms following TBEV infection that vary over time and across age groups. This study highlights the need for standardized symptom categorization and follow-up time for TBE sequelae studies.

## Teaser

Following tick-borne encephalitis virus infection, patients can experience a variety of neurological, neuropsychiatric, or other sequelae symptoms. Symptom prevalence changes over time and can differ between children and adults. Persistent and progressive sequelae merit further research.

Tick-borne encephalitis (TBE) is an infectious disease caused by the TBE virus (TBEV) that can result in signs and symptoms of central nervous system (CNS) and peripheral nervous system (PNS) inflammation with subsequent neurological damage [1]. TBEV infection is most often acquired by the bite of a tick (genus *Ixodes*) or occasionally through consumption of unpasteurized milk products [2–4]. There are 3 major subtypes of TBEV: European, Siberian, and Far Eastern [3, 5]. While the proportion of asymptomatic individuals is not precisely known, epidemiological data across all TBEV subtypes suggest that approximately 30% of infected individuals remain asymptomatic throughout the course of infection [6]. For the European subtype, approximately 74%–85% of infected patients experience a diphasic course, with an initial disease phase of nonspecific, flulike symptoms (1–8 days) followed by an asymptomatic period (1–20 days) before occurrence of signs and symptoms of nervous system involvement [6]. Clinical manifestations of TBEV infection include fever, headache, body aches, malaise, and a variety of neurological symptoms, indicative of meningitis, encephalitis, myelitis, radiculitis, or a combination thereof [6, 7]. Many patients with a diagnosis of TBE require hospitalization, and up to 15.5% of hospitalized patients require treatment in an intensive care unit [8].

TBE incidence has risen across Europe in recent years, along with its associated healthcare costs [9, 10]. Despite the availability of preventative measures, including safe and effective vaccines [1], TBE awareness is limited and TBE vaccination rates are low in many European countries [6]. Additional explanations for the rise in TBE incidence may include the geographic expansion of endemic areas and the extension of the transmission season, possibly driven by climate change (changes in humidity, temperature, and landscape); changes in human behavior, activities, or consumer demands; and the distribution/abundance of tick species and vector reservoirs [6, 11–14].

A prospective study in central Europe of 420 adults reported persistent symptoms, including headache (31.0%), memory/concentration disorders (28.8%), arthralgias/myalgias (13.8%), and emotional lability (13.6%), 2–7 years after TBE hospitalization [15]. A Swedish case-control study of adults reported that many had persistent neurological symptoms 2–15 years (median of 5.5 years) after TBE hospitalization [16]. TBE has also been associated with severe neurological sequelae, such as spinal cord injury, including paresis and bladder/bowel dysfunction [16–21]. The course and prognosis of sequelae following TBEV infection has been reported to depend on the infection route, TBEV subtype, concomitant diseases and medication, genetic predisposition, and age [3, 22–24]. While children are often reported to present with milder symptoms and a lower incidence of long-term morbid conditions than adults, there is some evidence of a higher burden of disease in children, with lifelong symptoms making a notable contribution to the overall burden, especially in the younger age groups [8, 25–27].

In this systematic review, we investigated the prevalence of persistent symptoms after hospital discharge in individuals with TBEV infection across multiple studies, populations, and follow-up time points to describe the current understanding of sequelae following TBE and TBEV infection in the published literature.

## METHODS

### Study Design

We searched PubMed, Embase, and Cochrane Library databases. Searches were conducted on 31 May 2024 and included publications of any language from 2007 to the present. The reference lists of included studies were searched for additional relevant studies. The systematic literature review inclusion criteria were (1) studies of adult and/or child participants with laboratory-confirmed TBEV infection (including non-CNS/PNS infection) or outbreak-linked TBEV infection and (2) either epidemiological, real-world studies or retrospective database studies. Studies meeting these inclusion criteria were rescreened for suitability for data analysis. For the analysis, we included studies with data within countries endemic for the European TBEV subtype, studies with laboratory-confirmed cases of TBE as defined by the European Centre for Disease Prevention and Control (ECDC), studies containing percentages or rates of known sequelae, and studies that provided information about patient signs and symptoms ≥1 day after hospital discharge.

Studies were excluded if they did not apply the ECDC TBE case definition or included insufficient information on laboratory methods to confirm the definition used. Studies and time points were also excluded from data analysis if (1) the study results were for a subset of the population (eg, only patients with magnetic resonance imaging), (2) the study reported on the same or overlapping cohorts as another study (duplicate data or double counting of patients), (3) the study did not primarily focus on TBEV infection or the outcomes were not specific to the TBEV-infected population, or (4) the study was a non–real-world evidence study (eg, randomized controlled trials) or a case report/series with <20 patients with TBE. Follow-up time points “at discharge” were also excluded in the analysis so that symptoms linked to “acute” illness would be distinguished from sequelae. All inclusion and exclusion criteria can be found in greater detail in Supplementary Table 1. We accepted the sequelae definitions implemented by each study and thoroughly documented these definitions.

### Data Analysis

Studies were categorized according to the duration of follow-up after discharge for patients with TBEV infection: ≤6, 7 to ≤12, or >12 months. Studies were also categorized into 3 groups: those that included only children, those that included only adults, and those that included both children and adults and did not explicitly distinguish between the age groups (“all ages”). Data were normalized using weighted mean percentages to account for different study sizes across time points and population types. Studies were first assessed by the proportion of patients with TBEV infections who reported any sequelae. Studies that reported percentages or rates of specific sequelae were further evaluated and organized into symptom groups. Sequelae symptoms were categorized into 3 groups: neurological, neuropsychiatric, and other symptoms. Forest plot summaries for TBEV infection sequelae proportions in each study were generated. The data heterogeneity (*I*^2^) was estimated by using a meta-analytic random-effects model in pediatric, adult, and all-age studies, separately.

## RESULTS

### Included Studies

**Figure 1.**
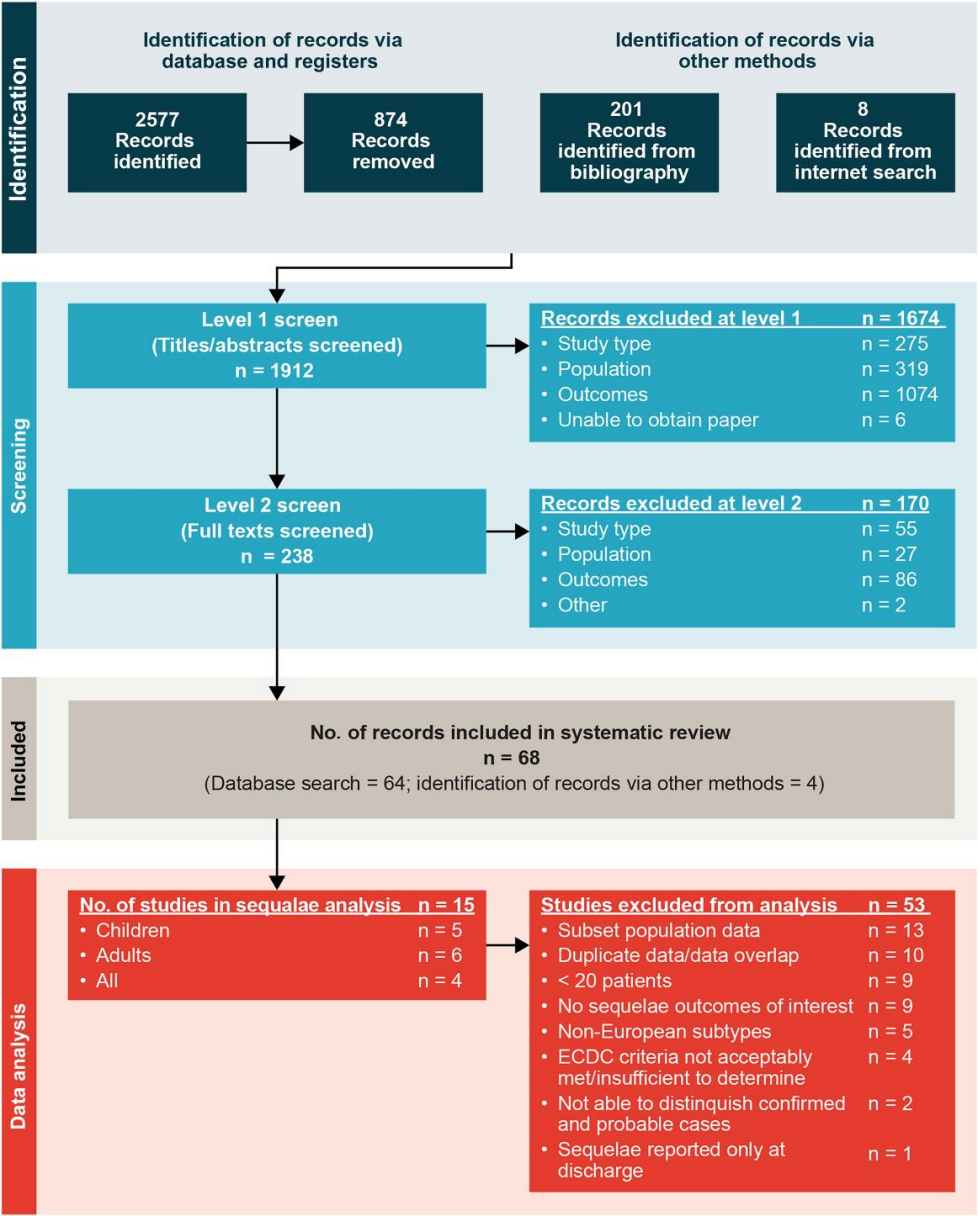
PRISMA (Preferred Reporting Items for Systematic Reviews and Meta-Analyses) diagram for systematic literature review, adapted from Page et al [28]. Abbreviation: ECDC, European Centre for Disease Prevention and Control.

A total of 1912 unique titles and abstracts were reviewed; 238 met the inclusion criteria and underwent full-text screening (Figure 1). After full-text screening, 68 publications were included, of which 15 remained after the data analysis inclusion criteria were applied: 6 studied sequelae in adults [17, 20, 21, 29–31], 5 in children, [25, 32–35] and 4 in all-age populations [27, 36–38] (Figure 1 and Table 1). Most studies (n = 13) included only hospitalized patients. Definitions of sequelae varied among studies (Supplementary Table 2). Follow-up times after hospital discharge among TBEV-infected patients varied within and between studies; therefore, data from the reviewed studies were grouped for analysis as ≤6 months (n = 6), 7 to ≤12 months (n = 3), or >12 months (n = 3) after hospital discharge.

**Table 1.** Details of Included Studies

Study Authors (Year)	Country	Study Period	Population	Total Participants, No.	TBE (CNS), No. (%)	Hospitalized Patients, No. (%)	Participants at Follow-up, No.	Time Points in Study	Participants With Sequelae, No. (%)	Age, y	Female Sex, No. (%)
Aregay et al (2024) [31]	Slovenia	NR	Adults (HOSP)	30	30 (100)	30 (100)	29	12 mo	13 (44.8)	Median (range): 59.5 (28–88)	15 (50)
Bogovič et al (2018) [29]	Slovenia	2007–2012	Adults (HOSP)	700	700 (100)	700 (100)	410	6 mo	170 (41.4)	Median (IQR): 53 (41–63)	305 (44)
								12 mo	131 (31.8)		
								24–84 mo	134 (32.8)		
Czupryna et al (2018) [17]	Poland	1993–2014	Adults (HOSP)	1072	1072 (100)	1072 (100)	1072	1 mo	221 (20.6)	Mean (SD): 44.1 (16.1)	404 (38)
Griška et al (2024) [30]	Lithuania	2018–2019	Adults (HOSP)	98	98 (100)	98 (100)	Follow-up 1: 77; follow-up 2: 61	Follow-up 1: 5 mo (median, 154 d); follow-up 2: 18 mo (median, 541 d)	Follow-up 1: 56 (72.7); follow-up 2: 43 (70.5)^a^	Mean (SD): 52.12 (16.62)	39 (39.8)
Lenhard et al (2016) [20]	Germany	2004–2014	Adults (HOSP)	111	111 (100)	111 (100)	59	12 mo	59 (100.0)	Median (range): 51 (17–75)	47 (42)
Misić Majerus et al (2009) [21]	Croatia	1995–2008	Adults (HOSP)	124	124 (100)	124 (100)	124	3 y	64 (51.6)	Range: 16–76	44 (35)
Fowler et al (2013) [32]	Sweden	2004–2008	Children (COMM)	66	66 (100)	46 (70)	42	24–84 y (mean, 4 y)	29 (69.0)	Mean (range): 10.8 (3–17)	26 (39)
Fritsch et al (2008) [33]	Austria	1981–2005	Children (HOSP)	116	116 (100)	116 (100)	116	6 mo	2 (1.7)	Median (range): 9.1 (0–15.5)	NR
Krawczuk et al (2020) [25]^b^	Poland	2004–2015	Children (HOSP)	69	69 (100)	69 (100)	22	<1 mo, >1 mo^c^	6 (27.3)	Mean (range): 12.16 (2.5–18)	31 (45)
Krbková et al (2015) [34]	Czech Republic	1993–2012	Children (HOSP)	170	153 (90.0)	170 (100)	170	3 mo to 3.2 y	24 (14.1) (cognitive/ neurological)	Range: 2–18	71 (42)
Majerus et al (2013) [35]	Croatia	1979–2011	Children (HOSP)	115	115 (100)	115 (100)	112	12 mo	35 (31.3)	Median (range): 10 (2–14)	27 (24)
Barp et al (2020) [36]	Italy	2000–2019	All ages (HOSP)	148	140 (94.6)	148 (100)	148	1 mo	52 (35.1)	Mean (range): 53 (13–81)	36 (24)
Nygren et al (2023) [27]	Germany	2018–2020	All ages (HOSP)	558	NR	471 (84.4)	523	<2, 2, 6, 12, and >18 mo	∼3 mo: 268 (51.2); ∼18 mo: 171 (32.7)^d^	Mean (SD): 48.7(19.9)	190 (36.3)
Rezza et al (2015) [37]	Italy	2000–2013	All ages (COMM)	367	307 (83.7)	NR	367	NR	60 (16.3)	Median (IQR): 56 (42–67)	110 (30)
Velay et al (2018) [38]^e^	France	2013–2016	All ages (HOSP)	54	46 (85.2)	53 (98)	27	15 d to 14 mo^f^	9 (33.3)	Mean (range): 48.6 (8–87)	16 (30)

Abbreviations: COMM, community data source; CNS, central nervous system; HOSP, hospitalised data source; IQR, interquartile range; NR, not reported; TBE, tick-borne encephalitis.

^a^The number 43 was provided following communication with the authors.

^b^For Krawczuk et al [38], adult data are not reported, as they overlapped with that of Czupryna et al [17].

^c^Data will be reported as ≤6 months.

^d^The percentage of patients with sequelae was calculated by subtracting the percentage of patients who were fully recovered at the time of the interview from 100%.

^e^One patient was community only and was not hospitalized.

^f^The median was 2 months, and the mean, 3 months. All reported sequelae specified in the text had onset at ≤6 months.

### Overview of Sequelae

Categorization of signs and symptoms differed among studies (Supplementary Table 2). The proportions of patients with sequelae across all time points varied between and within the different age groups: from 20.6% [17] to 100.0% [20, 21] for adults, 1.7% [33] to 69% [32] for children, and 16.3% [37] to 51.2% [27, 36] for studies that did not distinguish between adults and children (all ages). The definition of “adult” varied between studies. For example, Misić Majerus et al [21] studied patients aged 16–76 years, who were included in the adult analysis. In comparison, Krbková et al [34] studied patients aged 2–18 years, and was included in the analysis of children. Studies using a prospective design reported sequelae with a frequency range of 31.3%–100%. We observed high levels of data heterogeneity in the meta-analyses (*I*^2^ > 90%) of all studies, regardless of population type (Supplementary Figure 1). Nine studies reported the specific sequelae symptoms experienced [17, 27] [30, 32–36, 38]. In total, 79 different symptoms were reported across the 10 studies. Of these 79 symptoms, those that were similar were grouped together (eg, balance disorders and incoordination were combined, as were sleep disorders and insomnia), providing 44 groups of symptoms. These 44 groups were then categorized into 3 types of sequelae: neurological, neuropsychiatric, and other.

### Neurological Sequelae

**Figure 2.**
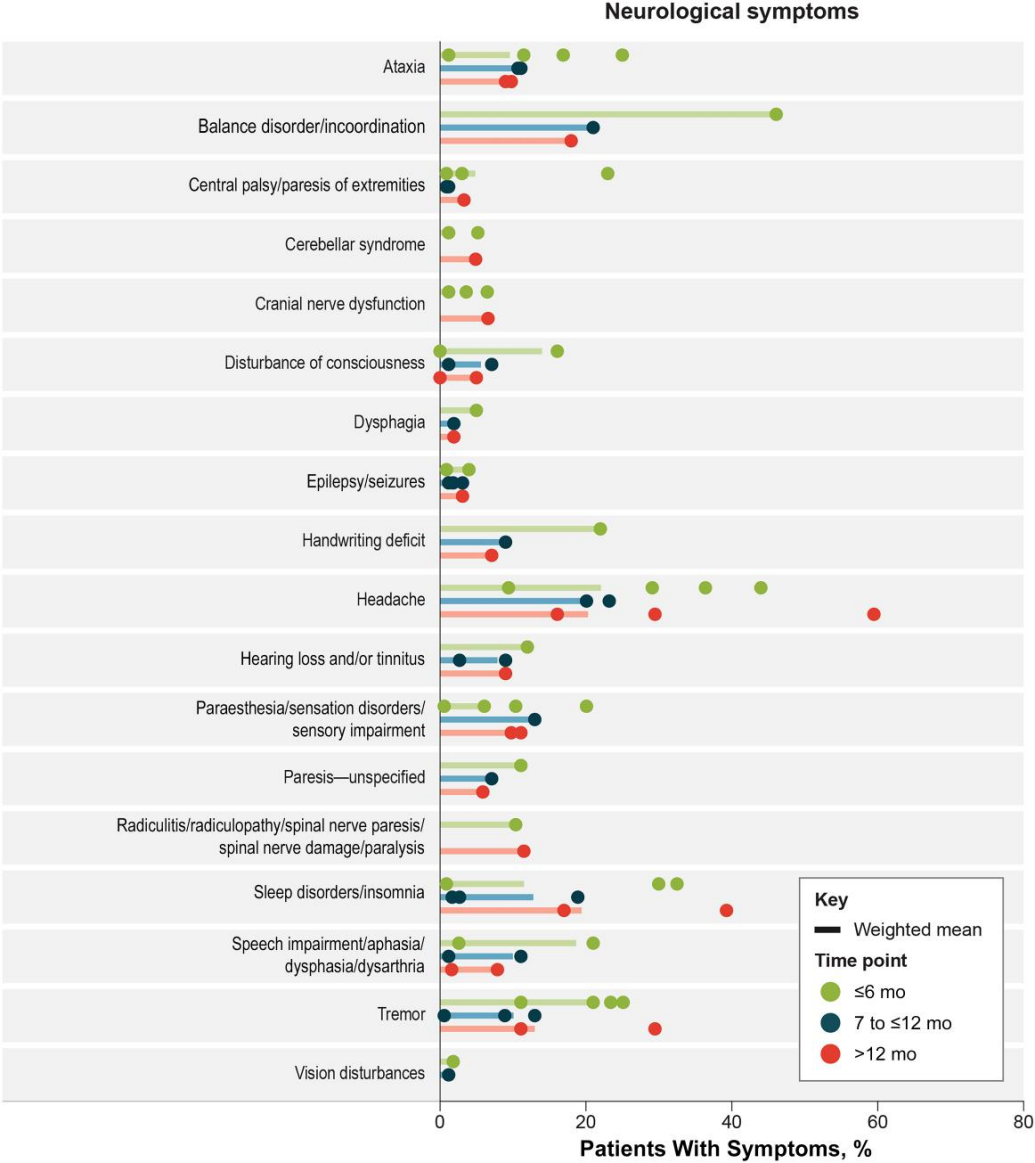
Neurological sequelae signs and symptoms. Symptoms with 1 data point include akinesia (2.03%) and neurological deficit (3.70%) (both at <6 months).

The proportion of patients with TBEV infection and neurological sequelae signs and symptoms after discharge are presented in Figure 2. In all, 20 neurological symptoms were reported. The most frequently reported (highest overall weighted mean proportions at any of the 3 time points) were balance disorders and headache. The proportion of patients with headache remained similar across all time points (approximately 20%). In contrast, the percentage of patients in some categories changed over time: the proportion of sleep disorders/insomnia after discharge increased over time (from 11.5% at ≤6 months to 19.4% at >12 months), while proportions decreased from the ≤6-month to the >12-month time point for balance disorders/incoordination (from 46.1% to 18.0%), handwriting deficits (from 22.0% to 7.1%), speech impairment (from 18.7% to 7.4%), paresis—unspecified (from 11.1% to 5.9%), and disturbance of consciousness (from 14.0% to 4.5%) (Supplementary Table 3).

### Neuropsychiatric Sequelae

**Figure 3.**
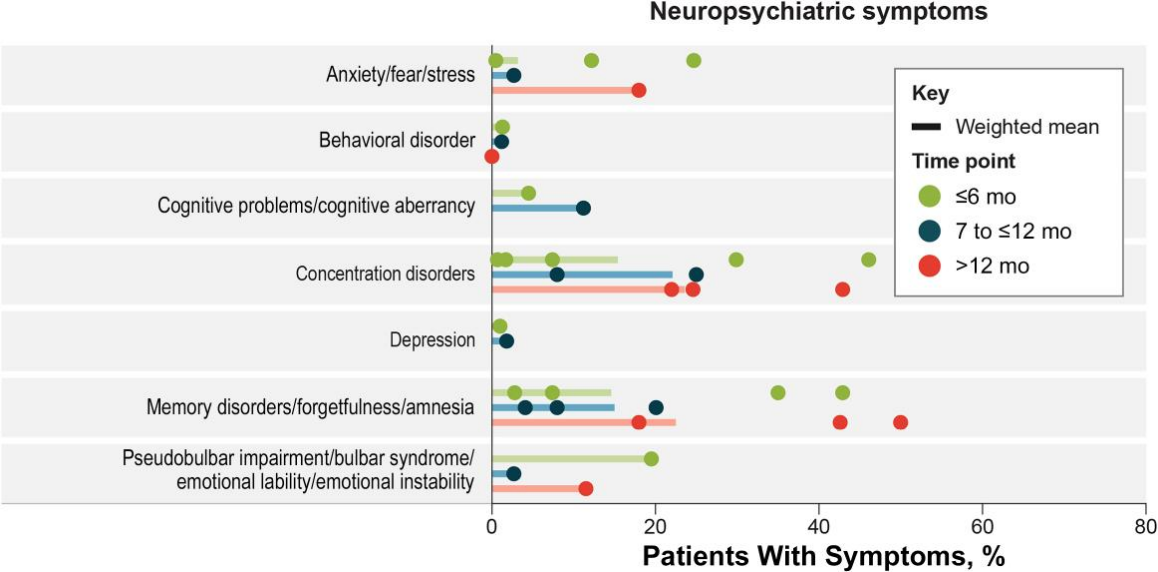
Neuropsychiatric sequelae signs and symptoms. Symptoms with 1 data point include attention problems (8.04%) at 7 to ≤12 months, psychotic (0.09%) and psychoorganic (0.19%) symptoms (both at <6 months).

The proportions of patients with TBEV infection and neuropsychiatric signs and symptoms after discharge are presented in Figure 3. The most commonly reported symptoms (highest overall weighted mean proportions at any of the 3 time points) were concentration disorders (15.5% at ≤6, 22.1% at 7 to ≤12, and 23.6% at >12 months) and memory disorders (13.6, 15.0%, and 22.5%, respectively), with reports of these symptoms increasing across all 3 time points. Other neuropsychiatric sequelae included anxiety, attention problems, behavioral disorders, cognitive problems, depression, psychotic symptoms, pseudobulbar impairment, and psychoorganic symptoms.

### Other Sequelae

**Figure 4.**
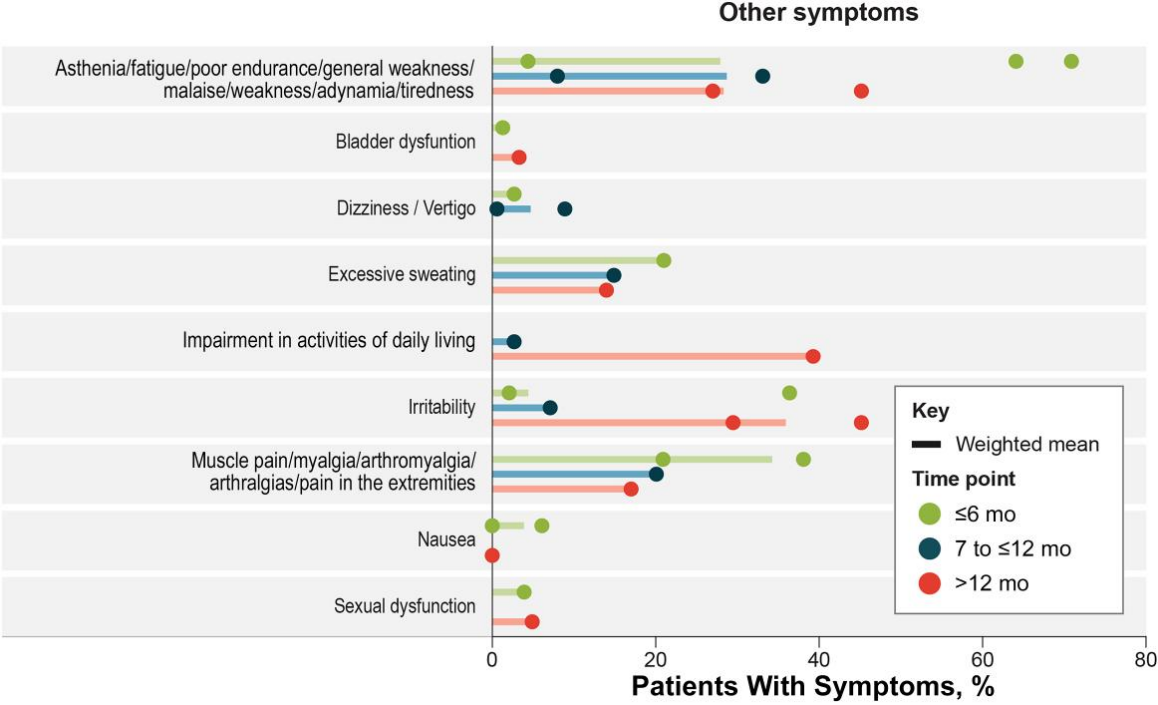
Other sequelae signs and symptoms. Symptoms with 1 data point include abdominal pain (4.05%) and muscle wasting/weakness (7.41%) (both at <6 months) and worsening of school grades (5.29%), amplified reflexes (8.04%), and electroencephalographic anomalies (1.76%) (at 7 to ≤12 months). For fatigue, note that Nygren et al [27] reported fatigue and general weakness separately; fatigue was selected for the purposes of categorization as it was reported by a higher proportion of patients.

Some studies reported symptoms among patients with TBE after discharge that did not fall into neurological or neuropsychiatric categories, including irritability, dizziness, asthenia, sexual dysfunction, and impairment of daily living (Figure 4). The most frequently reported other sequelae symptoms (highest overall weighted mean proportions at any of the 3 time points) were impaired activities of daily living, irritability, and muscle pain. Muscle pain decreased from ≤6 months to >12 months after discharge for all ages (from 34.3% to 17.0%). Irritability increased over time across all age groups from ≤6 months to >12 months after discharge (overall, from 4.4% to 35.9%). Impairment in activities of daily living increased from 2.7% to 39.3% after discharge in small follow-up cohorts of children at 7 to ≤12 and >12 months (Table 1 and Supplementary Table 3).

### Sequelae Among Children and Adults

Five of the studies reported sequelae in children after discharge, with prevalences ranging from 1.7% to 69.0%. This compared with the prevalence in adults, ranging from 20.6% to 100% (Table 1). The types of symptoms were reported in 3 pediatric studies, but patient numbers were small. Weighted percentages of sequelae by time point and age group can be found in Supplementary Table 3. Symptoms reported only in adults were bladder dysfunction, cerebellar syndrome, psychotic symptoms, psychoorganic symptoms, radiculitis, and sexual dysfunction. In contrast, attention problems, amplified reflexes, electroencephalographic anomalies, impairment in activities of daily living, and worsening of school grades were reported exclusively in children. In general, neurological symptoms were more commonly reported in adults. All age groups experienced headache as a persistent symptom but with a higher prevalence at >12 months (in 59.5% of children vs 29.5% of adults). Similarly, both adults and children experienced memory disorders that persisted and had a higher prevalence at >12 months (in 50.0% of children vs 42.6% of adults).

## DISCUSSION

To our knowledge this is the first study to comprehensively review published sequelae occurring after discharge in patients hospitalized with TBEV infection. The proportion of patients with nervous system manifestations of TBEV infection affected by sequelae following hospital discharge varied across studies, time points, and population types. Two of the most prominent sequelae symptoms reported consistently across all time points were headache and asthenia. Both were consistently reported for >20% of patients across all time points after discharge, highlighting them as prominent and persistent symptoms afflicting patients and suggesting that further studies are warranted to improve patient care. Other proportions of symptoms fluctuated over time. Decreases were reported in balance disorders, handwriting deficits, and muscle pain across time points, while other symptoms, like sleep disorders, anxiety, and memory disorders, increased over 12 months. Sequelae symptoms can significantly alter the overall quality of life for individuals following TBEV infection [39]. Sequelae after TBE should be routinely assessed at discharge and at different time points after discharge. Given the wide breadth of sequelae symptoms experienced by both children and adults, continued assessment can facilitate more successful rehabilitation and allow for tailored interventions specific to the needs of each individual patient [40].

TBEV and other causes of viral encephalitis can result in numerous persistent sequelae, including disorders of behavior, speech, and memory [41]. Insomnia and sleep disorders following inflammatory conditions of the CNS have been reported, particularly for viral encephalitis, though estimates of prevalence vary [42–45]. A European multicenter study from 2010 to 2017 reported that 32% of hospitalized patients with TBE had sleep disturbances after discharge [46]. The data in the current study are consistent with previous research and suggest a link between long-term disordered sleep and TBEV infection. Further research into the connection between TBE and sleep disorders may provide valuable insight on the cause and subsequently the treatment of TBEV infection sequelae. Chronic headache has similarly been reported following infection and viral encephalitis [41 , 47].

Interestingly, in this review, many of the sequelae reported in children with TBEV infection fell into the neuropsychiatric category. Viral meningitis and encephalopathy have similarly been associated with a variety of cognitive issues, including memory, learning, and concentration disorders, irritability, and anxiety [48–50]. The data presented here highlight the repercussions of direct TBE-acquired brain injury that may similarly indicate a connection between TBEV infections and neuropsychiatric disorders in children. Sequelae may negatively affect children's daily life, school performance, and other abilities critical to cognitive development [51, 52]. Little is known about the consequences of a TBEV infection on the developing brain, highlighting the need for future research on TBEV infection sequelae, especially in zchildren.

There is no standard case definition for sequelae. Long-term signs and symptoms vary and can be subjective (eg, anxiety/fear and irritability), and follow-up time points after discharge are not consistent; therefore, the synthesis of comprehensive information is limited. Some rarer sequelae may be reported only in case studies and may therefore not be captured in this review. Some sequelae were reported only in pediatric studies and may not have been recorded in adult studies, such as impaired activities of daily living; a lack of reporting does not necessarily mean that the symptom does not affect adults. Categorization of symptoms and reported proportions of sequelae may vary between hospitalized and nonhospitalized patients, who were analyzed together in the current study. Some studies used a prospective design, which may have resulted in a larger number of persistent symptoms being reported. In addition, categorization may vary between studies; for example, what is entered as “paresis—unspecified” in one study could be entered as “paresis of extremities” in another.

This data set was highly heterogenous among all study populations, regardless of age. There was a lack of information on patient comorbid conditions or immunization status, both of which have been associated with overall outcomes [53, 54]. Not all studies distinguished between TBEV infections with or without nervous system involvement. Because of the limited availability of reported outcomes, we grouped all CNS/PNS outcomes for the current study, but in the future it would be valuable to investigate sequalae by nervous system manifestation. In doing so, we have identified a need for clinical data on this topic, as the type and severity of nervous system manifestation can result in a variety of long-term outcomes [55–58] and highlighted an open area of research to determine whether these factors are associated with a higher risk of long-term disability or lower quality of life. Patients may appear across more than a single symptom category group, so we could not group patient data and compare across categories. Not all studies reported sequelae symptoms for all time points after discharge (eg, cognitive problems and behavioral disorders were assessed only at ≤6 months and 7 to ≤12 months); similarly, comparing between time points often necessitated comparing data from >1 study.

Despite its limitations, the current study also has several strengths. Sample sizes within individual studies remained consistent at all measured time points, indicating that patients who did not experience sequelae symptoms were not lost to follow-up. Weighted means were used to account for varying sample sizes between studies. This is among the first studies to provide a comprehensive overview of TBEV infection sequelae. This work further bolsters the need for common criteria for the assessment of TBE disease severity and standardized follow-up time points for evaluating sequelae and quality of life [59].

In conclusion, after TBEV infection, patients can experience a wide variety of neurological, neuropsychiatric, or other sequelae symptoms that have been reported at follow-ups more than a year after hospital discharge. While the proportion of some sequelae decreased over time, it remained constant or even increased for others. For example, sleep disorders were found to increase in prevalence over time and headache to persist for many patients. Better understanding of the persistent and progressive sequelae affecting patients after discharge, and the mechanisms behind their occurrence, can better inform future research and treatment options. The findings of this study also emphasize the need for standardized symptom categorization and follow-up time periods to increase comparability among future studies of TBE sequelae in both children and adults.

## Supplementary Material

ofaf317_Supplementary_Data

## Supplementary Data

Supplementary materials are available at *Open Forum Infectious Diseases* online. Consisting of data provided by the authors to benefit the reader, the posted materials are not copyedited and are the sole responsibility of the authors, so questions or comments should be addressed to the corresponding author.
