# Correction to: Clinical Spectrum and Dynamics of Sequelae Following Tick-Borne Encephalitis Virus Infection: A Systematic Literature Review

**DOI:** 10.1093/ofid/ofaf558

**Published:** 2025-09-11

This is a correction to: Kate Halsby, Liesl Gildea, Pingping Zhang, Frederick J Angulo, Andreas Pilz, Jennifer Moisi, Ann Colosia, Johann Sellner, Clinical Spectrum and Dynamics of Sequelae Following Tick-Borne Encephalitis Virus Infection: A Systematic Literature Review, *Open Forum Infectious Diseases*, Volume 12, Issue 6, June 2025, ofaf317, https://doi.org/10.1093/ofid/ofaf317

The graphical abstract was erroneously missed from the originally published version of the manuscript. This has now been added to the article.

