# Supplementary material for: Clinical Spectrum and Dynamics of Sequelae Following Tick-Borne Encephalitis Virus Infection: A Systematic Literature Review
Source: Open Forum Infect Dis. 2025 May 29;12(6):ofaf317. doi: 10.1093/ofid/ofaf317 (PMC12188206; doi:10.1093/ofid/ofaf317)
Supplement: ofaf317_Supplementary_Data [file ofaf317_supplementary_data.docx]

Supplemental Material For: Clinical Spectrum and Dynamics of Sequelae Following Tick-Borne Encephalitis Virus Infection: A Systematic Literature Review

# SUPPLEMENTAL FIGURE

Figure S1. Forest Plot of Data Heterogeneity


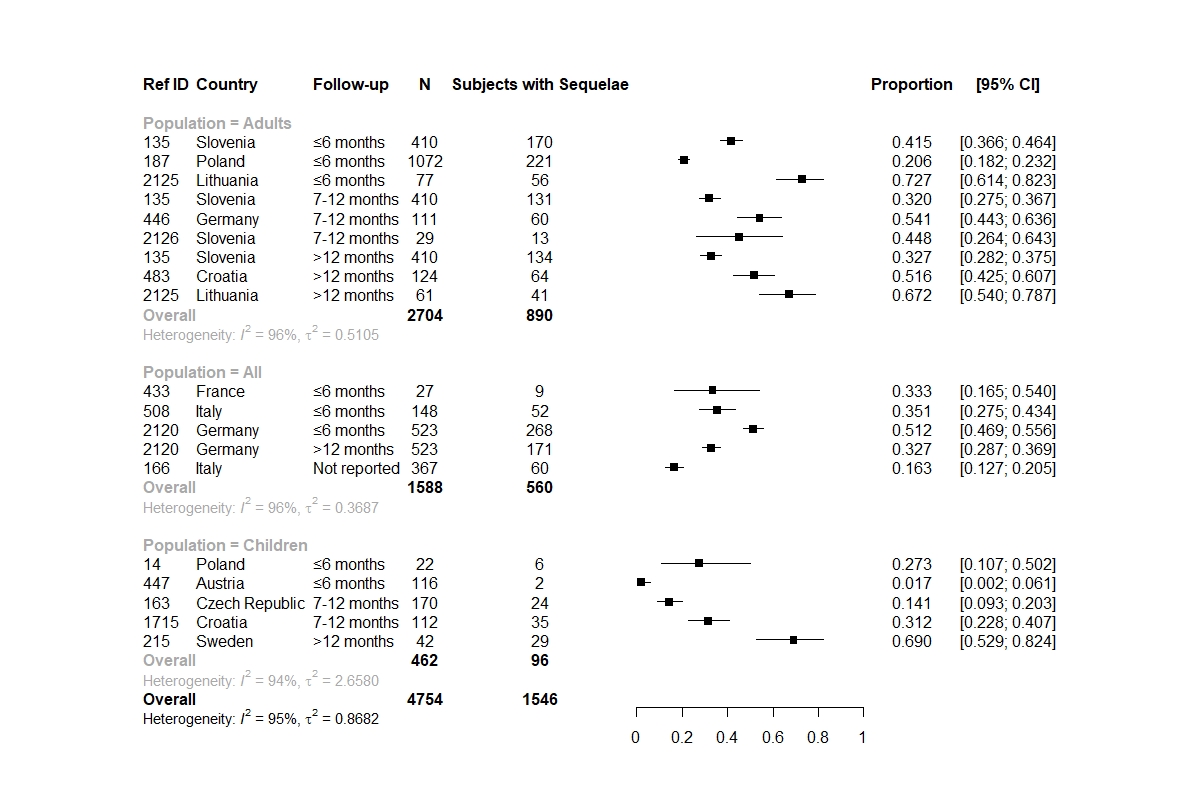


# SUPPLEMENTAL TABLES

Table S1. Inclusion and Exclusion Criteria

| Criterion | Included | Excluded |
| --- | --- | --- |
| Population | Individuals with laboratory-confirmed TBEV infection (including non-CNS infection) or outbreak-linked TBEV infection and sequelae, both adults and children  Regions where the European subtype of TBEV is prevalent | Individuals do not have TBEV infection  Individuals have TBE virus infection that is only clinically diagnosed (e.g., ICD coding) but without laboratory confirmation or without outbreak linkage to laboratory-confirmed case(s), or they have TBE virus infection that is not the focus of the publication |
| Interventions | None/any (i.e., do not exclude for intervention) | None |
| Comparators | None/any (i.e., do not exclude for comparators) | None |
| Outcomes | Known sequelae among TBE cases:  Proportions of patients experiencing aspects of the clinical spectrum of TBE virus infection  Proportion of patients who were:   - Hospitalised - Admitted to the ICU - Recovered - Had sequalae following CNS disease of TBEV infection - Died | Outcomes not specific to the TBEV-infected population (e.g., outcomes are for all meningitis, regardless of causative agent) |
| Study types | Epidemiological studies  Retrospective database studies  Real-world studies, including the following:   - Prospective observational studies - Retrospective studies   Robust SLRs or highly informative general review articles (to help identify additional primary studies) | Non–real-world evidence studies (e.g., randomised controlled trials)  Case reports  Case series |
| Language | Languages for which a translator is available | Languages for which it is difficult to find a translator^a^ |
| Time point | All time points beyond discharge | Follow-up time points collected “at discharge” |

CNS = central nervous system; ICD = International Classification of Diseases; ICU = intensive care unit; SLR = systematic literature review; TBEV = tick-born encephalitis virus.

^a^ Translators were identified for all non-English full-text articles included after title/abstract review.

Table S2. Criteria for Sequelae in Included Studies

| Reference | Country | Population | Criteria of sequela/postencephalitic syndrome |
| --- | --- | --- | --- |
| Krawczuk et al.^1^ | Poland | Children | - Early subjective conditions (< 1 month), such as headache and vertigo - Early mental conditions (< 1 month), such as memory impairment, loss of concentration, sleep disturbances, and depression and anxiety - Early neurological conditions (< 1 month), such as upper/lower limb paresis, cranial nerve paresis, and cerebellar syndrome - Late subjective conditions (≥ 1month), such as headache and vertigo - Late mental conditions (≥ 1 month), such as memory impairment, loss of concentration, sleep disturbances, and depression and anxiety - Late neurological conditions (≥ 1 month), such as upper/lower limb paresis, cranial nerve paresis, and cerebellar syndrome |
| Krbková et al.^2^ | Czech Republic | Children | Neurological symptoms affecting previous daily activities were considered as severe sequelae, whereas residual symptoms with no impact upon the quality of life were defined as mild or moderate. Subjective complaints such as headache or fatigue during the first 3 months after TBE were not classified as sequelae. |
| Fritsch et al.^3^ | Austria | Children | Not reported. The 2 patients with sequelae were reported as “Two patients (1.7%) had severe neurological sequelae 6 months after hospital discharge.” |
| Majerus et al.^4^ | Croatia | Children | Not reported |
| Aregay, 2024^5^ | Slovenia | Adults | The presence of subjective pre-defined TBE associated symptoms and/or objective neurological sequelae one year after acute phase were considered as unfavorable outcome, as described previously ^6^.  Patients without these symptoms were categorized as having favorable clinical outcomes.  From Bogovic et al.^6^:  Subjective symptoms (fatigue, headache, arthralgias and myalgias, memory and concentration disorders, emotional lability, sleep disorders, dizziness, etc.) fulfilling criteria for NOIS (new or increased symptoms) and as objective neurological signs (tremor, ataxia, cranial and spinal nerve pareses, etc.).  An unfavourable long-term clinical outcome (PES) was defined as the presence of ≥ 2 subjective symptoms fulfilling criteria for NOIS and/or ≥ 1 objective neurological sign at the 6-month follow-up or later. |
| Bogovič et al.^7^ | Slovenia | Adults | Sequelae of TBE were defined as subjective symptoms (e.g., fatigue, headache, arthralgias and myalgias, memory and concentration disorders, emotional lability, sleep disorders, dizziness) fulfilling criteria for new or increased symptoms and as objective neurological signs (e.g., tremor, ataxia, cranial and spinal nerve paresis) present at ≥ 6 months after the acute illness. No formal testing of cognitive function was performed. However, at the final visit, 2±7 years after acute illness, the presence of 6 nonspecific symptoms occurring within the preceding 4 weeks was determined and the SF-36 Health Survey was performed. |
| Czupryna et al.^8^ | Poland | Adults | Clinical symptoms during follow-up. Sequelae were defined as symptoms that persisted or appeared at least 1 month after the first hospitalisation (discharge from hospital) and affected patients’ life. |
| Fowler, 2013^9^ | Sweden | Adults | Not reported – states only that the persistence of 3 or more symptoms was reported at follow-up by 29 of 42 children. |
| Griška, 2024^10^ | Lithuania | Adults | Classified long-term sequelae and disability at two time-points using two distinct methods: first, registering objective neurological signs and subjective symptoms, second – using MRS (Modified Rank-in Score).  0. No symptoms at all  1. No disability despite minimal symptoms. Able to carry out all usual duties and activities  2. Slight disability. Able to look after own affairs without assistance, but unable to carry out all previous activities.  3. Moderate disability. Requiring some help, but able to walk without assistance  4. Moderately severe disability. Unable to walk without assistance, and substantial loss of independence but no need for constant assistance  5. Severe disability. Bedridden, incontinent and requiring constant nursing care  6. Death |
| Lenhard et al.^11^ | Germany | Adults | Functional outcome of meningoencephaloradiculitis and meningoencephalitis according to the modified Rankin scale at admission compared with long-term outcome as shift analysis by degree of disability. Modified Rankin scale: 0, no symptoms at all; 1, no significant disability—able to carry out all usual activities despite some symptoms; 2, slightly disabled—able to look after own affairs without assistance but unable to carry out all previous activities; 3, moderate disability—requires some help but able to walk unassisted; 4, moderately severe disability—unable to attend to own bodily needs without assistance; 5, severe disability—requires constant nursing care and attention, bedridden, incontinent; and 6, dead. |
| Misić Majerus et al.^12^ | Croatia | Adults | Based on the collected data, the patients are divided into 3 groups through a simple qualitative assessment of the effect of PES on their daily life habits and activities:   - Patients with mild, transient PES, without a significant effect on the quality of their daily life and with full recovery within 6 weeks - Patients with moderate PES, manifested by a significant effect on the quality of their daily life habits and activities, but without the need for adaptation, lasting more than 3 months - Patients with severe PES (including paresis, balance and movement coordination disorders, hearing and vision disorders), which require adaptation in their daily life |
| Rezza, 2015^13^ | Italy | Adults | Not reported |
| Barp et al.^14^ | Italy | All ages | Not reported |
| Nygren et al.^15^ | Germany | All ages | PES in mild form was defined as the presence of 2-3 subjective symptoms reported as due to TBE virus infection at ≥ 6 months after onset, following the definition in Bogovic et al.^6^. Because data were self-reported and, thus, with a risk toward overreporting of symptoms, we chose more conservative cutoffs with 4-5 subjective symptoms for moderate and ≥ 6 subjective symptoms for severe PES. |
| Velay, 2018^16^ | France | All ages | Not reported – states “incomplete recovery at last follow up.” |

PES = postencephalitic syndrome; TBE = tick-borne encephalitis.

Table S3. Weighted Percentage of Sequelae Symptoms for Adults and Children

| **Neurological Sequelae** | **Population** | **Months** | | | **Neuropsychiatric Sequelae** | **Population** | **Months** | | | **Other Sequela** | **Population** | **Months** | | | |
| --- | --- | --- | --- | --- | --- | --- | --- | --- | --- | --- | --- | --- | --- | --- | --- |
|  |  | **≤6** | **7-≤12** | **>12** |  |  | **≤6** | **7-≤12** | **>12** |  |  | **≤6** | **7-≤12** | | **>12** |
| **Akinesia** | **Adults** |  |  |  | **Anxiety / Fear /Stress (n)** | **Adults** | 2.09 |  | 18.03 | **Impairment of activities of daily living** | **Adults** |  |  |  | |
|  | **Children** |  |  |  |  | **Children** |  | 2.68 |  |  | **Children** |  | 2.68 | 39.29 | |
|  | **All ages** | 2.03 |  |  |  | **All ages** | 12.16 |  |  |  | **All ages** |  |  |  | |
|  | **Overall** | 2.03 |  |  |  | **Overall** | 3.24 | 2.68 | 18.03 |  | **Overall** |  | 2.68 | 39.29 | |
| **Ataxia** | **Adults** | 1.91 |  | 9.84 | **Attention problems** | **Adults** |  |  |  | **Worsening of school grades** | **Adults** |  |  |  | |
|  | **Children** |  | 10.71 |  |  | **Children** |  | 8.04 |  |  | **Children** |  | 5.29 |  | |
|  | **All ages** | 23.25 | 11.09 | 8.99 |  | **All ages** |  |  |  |  | **All ages** |  |  |  | |
|  | **Overall** | 9.78 | 11.02 | 9.08 |  | **Overall** |  | 8.04 |  |  | **Overall** |  | 5.29 |  | |
| **Balance disorder/**  **Incoordination** | **Adults** |  |  |  | **Behavioural disorder** | **Adults** | 1.30 |  | 0.00 | **Abdominal Pain** |  |  |  |  | |
|  | **Children** |  |  |  |  | **Children** |  | 1.18 |  |  | **Children** |  |  |  | |
|  | **All ages** | 46.08 | 21.03 | 17.97 |  | **All ages** |  |  |  |  | **All ages** | 4.05 |  |  | |
|  | **Overall** | 46.08 | 21.03 | 17.97 |  | **Overall** | 1.30 | 1.18 | 0.00 |  | **Overall** | 4.05 |  |  | |
| **Cerebellar syndrome** | **Adults** | 1.48 |  | 4.92 | **Cognitive problems / Cognitive aberrancy** | **Adults** | 4.48 |  |  | **Bladder dysfunction** | **Adults** | 1.30 |  | 3.28 | |
|  | **Children** |  |  |  |  | **Children** |  | 11.18 |  |  | **Children** |  |  |  | |
|  | **All ages** |  |  |  |  | **All ages** |  |  |  |  | **All ages** |  |  |  | |
|  | **Overall** | 1.48 |  | 4.92 |  | **Overall** | 4.48 | 11.18 |  |  | **Overall** | 1.30 |  | 3.28 | |
| **Disturbance of consciousness** | **Adults** | 0.00 |  | 0.00 | **Concentration disorders** | **Adults** | 3.57 |  | 24.59 | **Dizziness / Vertigo** | **Adults** | 2.71 |  |  | |
|  | **Children** |  | 1.18 |  |  | **Children** |  | 8.04 | 42.86 |  | **Children** |  | 4.61 |  | |
|  | **All ages** | 16.06 | 7.07 | 4.97 |  | **All ages** | 34.96 | 25.05 | 21.99 |  | **All ages** |  |  |  | |
|  | **Overall** | 14.00 | 5.63 | 4.45 |  | **Overall** | 15.54 | 22.05 | 23.64 |  | **Overall** | 2.71 | 4.61 |  | |
| **Dysphagia** | **Adults** |  |  |  | **Depression** | **Adults** | 1.03 |  |  | **Excessive sweating** | **Adults** |  |  |  | |
|  | **Children** |  |  |  |  | **Children** |  | 1.79 |  |  | **Children** |  |  |  | |
|  | **All ages** | 4.97 | 1.91 | 1.91 |  | **All ages** |  |  |  |  | **All ages** | 21.03 | 14.91 | 13.96 | |
|  | **Overall** | 4.97 | 1.91 | 1.91 |  | **Overall** | 1.03 | 1.79 |  |  | **Overall** | 21.03 | 14.91 | 13.96 | |
| **Epilepsy / seizures** | **Adults** |  |  |  | **Memory disorders / Forgetfulness / Amnesia / Memory and/or concentration disorder** | **Adults** | 5.48 |  | 42.62 | **Asthenia / Fatigue / Poor endurance / General weakness / Malaise / Weakness / Adynamia / Tiredness** | **Adults** | 4.38 |  |  | |
|  | **Children** | 0.86 | 1.42 |  |  | **Children** |  | 5.67 | 50.00 |  | **Children** |  | 8.04 | 45.24 | |
|  | **All ages** | 4.02 | 3.06 | 3.06 |  | **All ages** | 26.93 | 20.08 | 17.97 |  | **All ages** | 65.57 | 33.08 | 26.96 | |
|  | **Overall** | 3.44 | 2.48 | 3.06 |  | **Overall** | 13.59 | 15.03 | 22.52 |  | **Overall** | 27.94 | 28.66 | 28.32 | |
| **Handwriting deficit** | **Adults** |  |  |  | **Pseudo-bulbar impairment/ Bulbar syndrome / Emotional lability / Emotional instability** | **Adults** | 19.48 |  | 11.48 | **Irritability** | **Adults** | 4.44 |  | 29.51 | |
|  | **Children** |  |  |  |  | **Children** |  | 2.68 |  |  | **Children** |  | 7.14 | 45.24 | |
|  | **All ages** | 21.99 | 8.99 | 7.07 |  | **All ages** |  |  |  |  | **All ages** |  |  |  | |
|  | **Overall** | 21.99 | 8.99 | 7.07 |  | **Overall** | 19.48 | 2.68 | 11.48 |  | **Overall** | 4.44 | 7.14 | 35.92 | |
| **Headache** | **Adults** | 11.23 |  | 29.51 | **Psychotic symptoms** | **Adults** | 0.09 |  |  | **Muscle pain / Myalgia / Arthromyalgia / Arthralgias / Pain in the extremities (n)** | **Adults** |  |  |  | |
|  | **Children** |  | 23.21 | 59.52 |  | **Children** |  |  |  |  | **Children** |  |  |  | |
|  | **All ages** | 40.69 | 20.08 | 16.06 |  | **All ages** |  |  |  |  | **All ages** | 34.28 | 20.08 | 17.02 | |
|  | **Overall** | 22.09 | 20.63 | 20.29 |  | **Overall** | 0.09 |  |  |  | **Overall** | 34.28 | 20.08 | 17.02 | |
| **Hearing loss and/or tinnitus** | **Adults** |  |  |  | **Psychoorganic symptoms** | **Adults** | 0.19 |  |  | **Muscle wasting /weakness** | **Adults** |  |  |  | |
|  | **Children** |  | 2.68 |  |  | **Children** |  |  |  |  | **Children** |  |  |  | |
|  | **All ages** | 12.05 | 8.99 | 8.99 |  | **All ages** |  |  |  |  | **All ages** | 7.41 |  |  | |
|  | **Overall** | 12.05 | 7.87 | 8.99 |  | **Overall** | 0.19 |  |  |  | **Overall** | 7.41 |  |  | |
| **Neurological deficit** | **Adults** |  |  |  |  |  |  |  |  | **Nausea** | **Adults** | 0.00 |  | 0.00 | |
|  | **Children** |  |  |  |  |  |  |  |  |  | **Children** |  |  |  | |
|  | **All ages** | 3.70 | 0.00 | 0.00 |  |  |  |  |  |  | **All ages** | 6.08 |  |  | |
|  | **Overall** | 3.70 |  |  |  |  |  |  |  |  | **Overall** | 4.00 |  | 0.00 | |
| **Paraesthesia / Sensation disorders / Sensory impairment** | **Adults** | 1.22 |  | 9.84 |  |  |  |  |  | **Sexual dysfunction** | **Adults** | 3.90 |  | 4.92 | |
|  | **Children** |  |  |  |  |  |  |  |  |  | **Children** |  |  |  | |
|  | **All ages** | 16.99 | 13.00 | 11.09 |  |  |  |  |  |  | **All ages** |  |  |  | |
|  | **Overall** | 7.03 | 13.00 | 10.96 |  |  |  |  |  |  | **Overall** | 3.90 |  | 4.92 | |
| **Cranial nerve dysfunction** | **Adults** | 1.57 |  | 6.56 |  |  |  |  |  | **Amplified reflexes** | **Adults** |  |  |  | |
|  | **Children** |  |  |  |  |  |  |  |  |  | **Children** |  | 8.04 |  | |
|  | **All ages** | 3.70 |  | 0.00 |  |  |  |  |  |  | **All ages** |  |  |  | |
|  | **Overall** | 1.56 |  | 6.56 |  |  |  |  |  |  | **Overall** |  | 8.04 |  | |
| **Central palsy/paresis of extremities** | **Adults** | 2.96 |  | 3.28 |  |  |  |  |  | **Electroencephalography anomalies** | **Adults** |  |  |  | |
|  | **Children** | 0.86 | 1.06 |  |  |  |  |  |  |  | **Children** |  | 1.76 |  | |
|  | **All ages** | 22.97 |  | 0.00 |  |  |  |  |  |  | **All ages** |  |  |  | |
|  | **Overall** | 4.88 | 1.06 | 3.28 |  |  |  |  |  |  | **Overall** |  | 1.76 |  | |
| **Paresis-unspecified** | **Adults** |  |  |  |  |  |  |  |  |  |  |  |  |  | |
|  | **Children** |  |  |  |  |  |  |  |  |  |  |  |  |  | |
|  | **All ages** | 11.09 | 7.07 | 5.93 |  |  |  |  |  |  |  |  |  |  | |
|  | **Overall** | 11.09 | 7.07 | 5.93 |  |  |  |  |  |  |  |  |  |  | |
| **Radiculitis / Radiculopathy / Spinal nerve paresis / Spinal nerve damage / Paralysis** | **Adults** | 10.39 |  | 11.48 |  |  |  |  |  |  |  |  |  |  | |
|  | **Children** |  |  |  |  |  |  |  |  |  |  |  |  |  | |
|  | **All ages** |  |  |  |  |  |  |  |  |  |  |  |  |  | |
|  | **Overall** | 10.39 |  | 11.48 |  |  |  |  |  |  |  |  |  |  | |
| **Sleep disorders / Insomnia** | **Adults** | 3.05 |  | 39.34 |  |  |  |  |  |  |  |  |  |  | |
|  | **Children** |  | 2.13 |  |  |  |  |  |  |  |  |  |  |  | |
|  | **All ages** | 30.02 | 18.93 | 17.02 |  |  |  |  |  |  |  |  |  |  | |
|  | **Overall** | 11.48 | 13.04 | 19.35 |  |  |  |  |  |  |  |  |  |  | |
| **Speech impairment / Aphasia / Dysphasia / Dysarthria** | **Adults** | 2.60 |  | 1.64 |  |  |  |  |  |  |  |  |  |  | |
|  | **Children** |  | 1.18 |  |  |  |  |  |  |  |  |  |  |  | |
|  | **All ages** | 21.03 | 11.09 | 8.03 |  |  |  |  |  |  |  |  |  |  | |
|  | **Overall** | 18.67 | 8.66 | 7.36 |  |  |  |  |  |  |  |  |  |  | |
| **Tremor** | **Adults** | 23.38 |  | 29.51 |  |  |  |  |  |  |  |  |  |  | |
|  | **Children** |  | 3.90 |  |  |  |  |  |  |  |  |  |  |  | |
|  | **All ages** | 21.49 | 13.00 | 11.09 |  |  |  |  |  |  |  |  |  |  | |
|  | **Overall** | 21.68 | 9.81 | 13.01 |  |  |  |  |  |  |  |  |  |  | |
| **Vision disturbances** | **Adults** |  |  |  |  |  |  |  |  |  |  |  |  |  | |
|  | **Children** |  | 1.18 |  |  |  |  |  |  |  |  |  |  |  | |
|  | **All ages** | 2.03 |  |  |  |  |  |  |  |  |  |  |  |  | |
|  | **Overall** | 2.03 | 1.18 |  |  |  |  |  |  |  |  |  |  |  | |

# References

1. Krawczuk K, Czupryna P, Pancewicz S, Ołdak E, Moniuszko-Malinowska A. Comparison of tick-borne encephalitis between children and adults-analysis of 669 patients. *J Neurovirol.* 2020;26(4):565-571. doi:<http://dx.doi.org/10.1007/s13365-020-00856-x>.

2. Krbková L, Štroblová H, Bednářová J. Clinical course and sequelae for tick-borne encephalitis among children in South Moravia (Czech Republic). *Eur J Pediatr.* 2015;174(4):449-458. doi:<http://dx.doi.org/10.1007/s00431-014-2401-8>.

3. Fritsch P, Gruber-Sedlmayr U, Pansi H, et al. Tick-borne encephalitis in Styrian children from 1981 to 2005: a retrospective study and a review of the literature. *Acta Paediatr.* 2008;97(5):535-538. doi:<http://dx.doi.org/10.1111/j.1651-2227.2008.00763.x>.

4. Majerus LM, Sabol Z, Mršić MT, Bujić N, Rode OD, Sabljić ER. Post-encephalitic syndrome in children with tick-borne encephalitis. *Paediatria Croatica.* 2013;57(1):61-66.

5. Aregay A, Slunečko J, Bogovic P, et al. Poor virus-specific T-cell responses early after tick-borne encephalitis virus infection correlate with disease severity. *Emerg Microbes Infect.* 2024;13(1):2317909. doi:<http://dx.doi.org/10.1080/22221751.2024.2317909>.

6. Bogovic P, Stupica D, Rojko T, et al. The long-term outcome of tick-borne encephalitis in Central Europe. *Ticks Tick Borne Dis.* 2018;9(2):369-378. doi:<http://dx.doi.org/10.1016/j.ttbdis.2017.12.001>.

7. Bogovič P, Lusa L, Stupica D, et al. Impact of pre-existing treatment with statins on the course and outcome of tick-borne encephalitis. *PLoS One.* 2018;13(10):e0204773. doi:<http://dx.doi.org/10.1371/journal.pone.0204773>.

8. Czupryna P, Grygorczuk S, Krawczuk K, et al. Sequelae of tick-borne encephalitis in retrospective analysis of 1072 patients. *Epidemiol Infect.* 2018;146(13):1663-1670. doi:<http://dx.doi.org/10.1017/s0950268818002005>.

9. Fowler Å, Forsman L, Eriksson M, Wickström R. Tick-borne encephalitis carries a high risk of incomplete recovery in children. *J Pediatr.* 2013;163 2:555-560.

10. Griška V, Pranckevičienė A, Pakalnienė J, et al. Long-term neurological and neurocognitive impairments after tick-borne encephalitis in Lithuania - a prospective study. *Infect Dis (Lond).* 2024;56(9):732-742. doi:<http://dx.doi.org/10.1080/23744235.2024.2346793>.

11. Lenhard T, Ott D, Jakob NJ, et al. Predictors, neuroimaging characteristics and long-term outcome of severe European tick-borne encephalitis: a prospective cohort study. *PLoS One.* 2016;11(4):e0154143. doi:<http://dx.doi.org/10.1371/journal.pone.0154143>.

12. Misić Majerus L, Daković Rode O, Ruzić Sabljić E. [Post-encephalitic syndrome in patients with tick-borne encephalitis]. *Acta Med Croatica.* 2009;63(4):269-278.

13. Rezza G, Farchi F, Pezzotti P, et al. Tick-borne encephalitis in north-east Italy: a 14-year retrospective study, January 2000 to December 2013. *Euro Surveill.* 2015;20(40). doi:<http://dx.doi.org/10.2807/1560-7917.Es.2015.20.40.30034>.

14. Barp N, Trentini A, Di Nuzzo M, Mondardini V, Francavilla E, Contini C. Clinical and laboratory findings in tick-borne encephalitis virus infection. *Parasite Epidemiol Control.* 2020;10:e00160. doi:<http://dx.doi.org/10.1016/j.parepi.2020.e00160>.

15. Nygren TM, Pilic A, Böhmer MM, Wagner-Wiening C, Wichmann O, Hellenbrand W. Recovery and sequelae in 523 adults and children with tick-borne encephalitis in Germany. *Infection.* 2023:1-9.

16. Velay A, Solis M, Kack-Kack W, et al. A new hot spot for tick-borne encephalitis (TBE): a marked increase of TBE cases in France in 2016. *Ticks Tick Borne Dis.* 2018;9(1):120-125. doi:<http://dx.doi.org/10.1016/j.ttbdis.2017.09.015>.
